# A novel nomogram to predict hemorrhagic transformation in ischemic stroke patients after intravenous thrombolysis

**DOI:** 10.3389/fneur.2022.913442

**Published:** 2022-09-08

**Authors:** Miaomiao Yang, Wei Zhong, Wenhui Zou, Jingzi Peng, Xiangqi Tang

**Affiliations:** ^1^Department of Neurology, The Second Xiangya Hospital of Central South University, Changsha, China; ^2^Department of Neurology, The First Affiliated Hospital of Shaoyang University, Shaoyang, China

**Keywords:** ischemic stroke, intravenous thrombolysis, nomogram, hemorrhagic transformation, predictors

## Abstract

**Background:**

Hemorrhagic transformation (HT) is the most serious complication of ischemic stroke patients after intravenous thrombolysis and leads to a poor clinical prognosis. This study aimed to determine the independent predictors associated with HT in stroke patients with intravenous thrombolysis and to establish and validate a nomogram that combines with predictors to predict the probability of HT after intravenous thrombolysis in patients with ischemic stroke.

**Method:**

This study enrolled ischemic stroke patients with intravenous thrombolysis from December 2016 to June 2022. All the patients were divided into training and validation cohorts. The nomogram was composed of the significant predictors for HT in the training cohort as obtained by the multivariate logistic regression analysis. The area under the receiver operating characteristic curve was used to assess the discriminative performance of the nomogram. The calibration performance of the nomogram was assessed by the Hosmer–Lemeshow goodness-of-fit test and calibration plots. Decision curve analysis was used to test the clinical validity of the nomogram.

**Results:**

A total of 394 patients with intravenous thrombolysis were enrolled in the study. In the training cohort (*n* = 257), 45 patients had HT after intravenous thrombolysis. Multivariate logistic regression analysis demonstrated early infarct signs (OR, 7.954; 95% CI, 3.553-17.803; *P* < 0.001), NIHSS scores (OR, 1.110; 95% CI, 1.054-1.168; *P* < 0.001), uric acid (OR, 0.993; 95% CI, 0.989–0.997; *P* = 0.001), and albumin-to-globulin ratio (OR, 0.109; 95% CI, 0.023–0.508; *P* = 0.005) were independent predictors for HT and constructed the nomogram. In the training and validation cohorts, the AUC of the nomogram was 0.859 and 0.839, respectively. The Hosmer–Lemeshow goodness-of-fit test and calibration plot showed good concordance between predicted and observed probability in the training and validation cohorts. Decision curve analysis indicated that the nomogram was significantly useful for predicting HT in the training and further confirmed in the validation cohort.

**Conclusion:**

This study proposes a novel and practical nomogram based on early infarct signs, NIHSS scores, uric acid, and albumin-to-globulin ratio that can well predict the probability of HT after intravenous thrombolysis in patients with ischemic stroke.

## Introduction

Ischemic stroke is the main cause of long-term disability and mortality worldwide (1, 2). Currently, intravenous thrombolysis with alteplase is the preferred method for patients with ischemic stroke within 4.5 h after onset (3). Hemorrhagic transformation (HT), especially symptomatic intracranial hemorrhage (SICH), is the most serious complication of intravenous thrombolysis that could lead to an increased probability of early neurological deterioration, severe disability, and death (4, 5). Therefore, assessing the risk of HT after intravenous thrombolysis in patients with ischemic stroke may help to improve clinical outcomes.

In recent years, several predictive scores have been reported to predict the risk of HT after intravenous thrombolysis, including the HAT score based on NIHSS score, glucose level, extent of hypodensity, and history of diabetes (6), SPAN-100 score based on age and NIHSS score (7), SENDA score based on age, early infarct signs, hyperdense cerebral artery sign, NIHSS score, and glucose level (8), and STARTING-SICH nomogram based on systolic blood pressure, hyperdense artery sign, current infarction sign, glucose, onset-to-treatment time, age, NIHSS scores, oral anticoagulant or aspirin or aspirin plus clopidogrel (9). Most of these studies were mainly focused on SICH after intravenous thrombolysis in Western patients with ischemic stroke. Yet, both symptomatic and asymptomatic intracerebral hemorrhage could lead to poor clinical outcomes (10, 11). In addition, previous studies reported that Asian patients with ischemic stroke have a higher risk of HT after intravenous thrombolysis compared with Western patients (12, 13). Several prognostic scores or nomograms for Asian stroke patients have been proposed to predict the risk of HT after intravenous thrombolysis in the past few years (14–16), but the effect of baseline neuroimaging or laboratory variables on HT after intravenous thrombolysis in these studies has been ignored. And nomograms may have better predictive performance than prognostic scores (9).

The nomogram is a graphical statistical tool that can assess and calculate the probability of a special clinical outcome for patients by using a continuous score, which has been used as a predictive method in stroke in recent years (9, 17). Therefore, the current study aimed to determine the independent predictors associated with HT in stroke patients with intravenous thrombolysis and to establish and validate a nomogram that combines neuroimaging and laboratory variables to predict the probability of HT after intravenous thrombolysis in patients with ischemic stroke.

## Materials and methods

### Study design and data source

This retrospective cohort study was conducted in accordance with the Declaration of Helsinki and approved by the Ethics Committee of the Second Xiangya Hospital of Central South University. The review board waived written informed consent due to the retrospective nature of the study.

In this study, we continuously enrolled patients diagnosed with ischemic stroke from December 2016 to June 2022 at the Second Xiangya Hospital of Central South University. Inclusion patients satisfied the criteria as follows: (1) age ≥ 18 years; (2) ischemic stroke was diagnosed with persistent neurological impairment and without any type of intracranial hemorrhage on non-contrast computed tomography (NCCT); (3) onset-to-treatment time <4.5 h; (4) patients received rt-PA intravenous thrombolysis; (5) diagnosis of with or without hemorrhagic transformation (HT) confirmed by non-contrast computed tomography (NCCT) or magnetic resonance imaging (MRI) within 22–36 h after rt-PA treatment. Patients who met the following criteria were excluded: (1) diagnosis of stroke mimics; (2) treatment with intra-arterial thrombolysis or endovascular thrombectomy after intravenous thrombolysis; (3) lack of complete data on all variables. All enrolled patients were divided into the training cohort and validation cohort based on the patients' years of diagnosis. The training cohort enrolled patients from December 2016 to December 2020. The validation cohort enrolled patients from January 2021 to June 2022. To avoid exposing patients' privacy, their identities were removed from the whole dataset before analysis.

### Clinical data collection

Baseline characteristics include demographic data (including age and gender), clinical data (including the history of hypertension, atrial fibrillation, diabetes mellitus, hyperlipidemia, previous stroke, smoking, drinking, and current use of anticoagulants or antiplatelet agents), early infarct signs on computed tomography (CT), onset-to-treatment time (OTT), National Institutes of Health Stroke Scale (NIHSS) scores, systolic/diastolic blood pressure, and laboratory data [blood glucose level, white blood cell (WBC) counts, neutrophil-to-lymphocyte ratio (NLR), platelet, prothrombin time (PT), activated partial thromboplastin time (APTT), fibrinogen, uric acid, albumin-to-globulin ratio (AGR), triglycerides, high-density lipoprotein (HDL), and low-density lipoprotein (LDL)] were collected for patients at admission. The hemorrhagic transformation (HT) was defined as any type of intracranial hemorrhage that was detected on follow-up CT or magnetic resonance imaging (MRI) within 22–36 h after intravenous thrombolysis, according to the criteria of the European Cooperative Acute Stroke Study II (18). All images were judged by two experienced neurologists blinded to the clinical data and final diagnosis.

### Statistical analysis

Continuous variables were described as mean ± SD or median (interquartile range, IQR), and categorical variables were expressed as frequency (percentage). The Student *t*-test or non-parametric Mann-Whitney *U* test was used for continuous variables, and the Chi-square test or Fisher's exact test was used for categorical variables. Variables with a *P*-value of <0.05 in the univariate analysis were included in the multivariate logistic regression analysis. Collinearity between each variable was assessed by the tolerance (<0.2 being considered significant) and variation inflation factors (>5 being considered significant). Finally, the odds ratio (OR) and 95% confidence interval (CI) of each variable were calculated by the multivariate logistic regression analysis.

A novel nomogram was used to establish the prediction model, which is based on the significant predictors of HT by the multivariate logistic regression analysis with the forward-section method. The area under the receiver operating characteristic curve (AUC–ROC) was used to assess the discriminative performance of the nomogram in the training cohort and validation cohort. The calibration performance of the nomogram in the training cohort and validation cohort was tested by using the Hosmer–Lemeshow goodness-of-fit test and a calibration plot with bootstraps of 1,000 resample, which described the concordance between the predicted and observed probabilities.

Decision curve analysis (DCA), a method for assessing the utility of prediction models, was further used to estimate the clinical validity of the nomogram in the training and validation cohorts. A detailed description of DCA was previously reported (19). Statistical analysis was performed using the statistical software IBM SPSS (version 26.0) and STATA (version 15.1). The significance level was set at a two-tailed *P* < 0.05.

## Results

**Figure 1:**
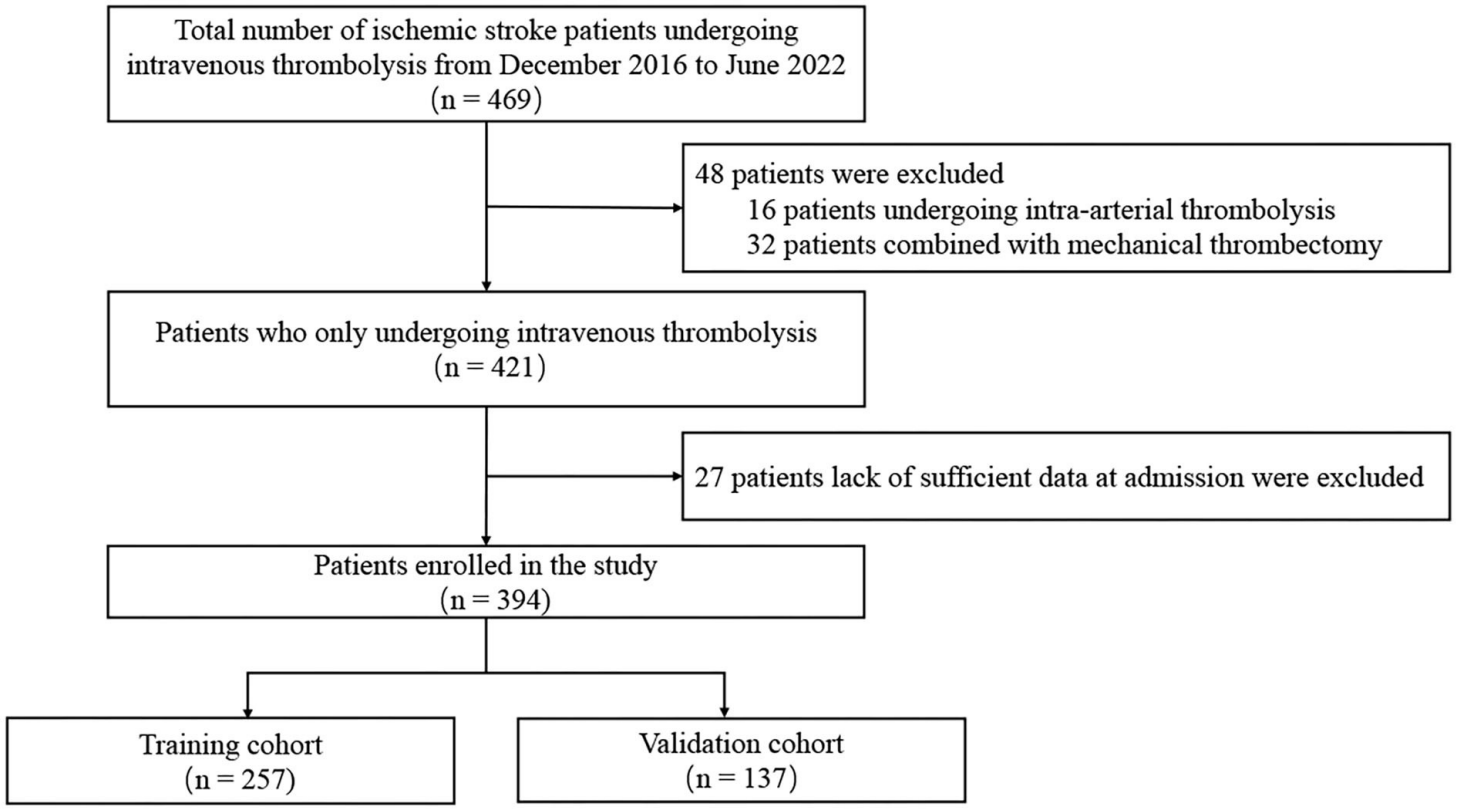
Flow chart of patient inclusion.

The flow chart of patient selection is shown in Figure 1. A total of 469 patients with ischemic stroke received rt-PA intravenous thrombolysis treatment. Patients treated with intra-arterial thrombolysis (*n* = 16) or endovascular thrombectomy (*n* = 32) and lack of complete data (*n* = 27) were excluded. Finally, 394 patients were included in the study and divided into the training cohort (*n* = 257) and validation cohort (*n* = 137) for further analysis. The median age of all patients was 65 (55–73) and 143 (36.3%) patients were female. Detailed information about the baseline characteristics of all patients is exhibited in Table 1. No significant differences in variables were observed between the training cohort and the validation cohort.

**Table 1:** Comparison of baseline characteristics between the training cohort and validation cohort.

**Variable**	**All patients (*n =* 394)**	**Training cohort (*n =* 257)**	**Validation cohort (*n =* 137)**	***P*-value**
**Demographic data**				
Age (years), median (IQR)	65 (55–73)	66 (55–74)	64 (55–73)	0.281
Female, *n* (%)	143 (36.3)	93 (36.2)	50 (36.5)	0.951
**Vascular risk factors**, ***n*** **(%)**				
Hypertension	265 (67.3)	176 (68.5)	89 (65.0)	0.478
Atrial fibrillation	120 (30.5)	74 (28.8)	46 (33.6)	0.326
Diabetes mellitus	95 (24.1)	62 (24.1)	33 (24.1)	0.993
Hyperlipidemia	32 (8.1)	22 (8.6)	10 (7.3)	0.663
Previous stroke	56 (14.2)	38 (14.8)	18 (13.1)	0.656
History of smoking	157 (39.8)	102 (39.7)	55 (40.1)	0.930
History of drinking	69 (17.5)	39 (15.2)	30 (21.9)	0.095
Antiplatelet agents	28 (7.1)	19 (7.4)	9 (6.6)	0.762
Anticoagulants	63 (16.0)	40 (15.6)	23 (16.8)	0.752
**Baseline data**				
OTT (min), median (IQR)	179 (134–227)	180 (140–234)	171 (130–215)	0.074
Early infarct signs, *n* (%)	103 (26.1)	67 (26.1)	36 (26.3)	0.964
NIHSS scores, median (IQR)	6 (3–13)	6 (3–13)	5.5 (3–13)	0.292
SBP (mmHg), mean ± SD	154 ± 25	156 ± 25	152 ± 25	0.156
DBP (mmHg), mean ± SD	88 ± 15	88 ± 15	87 ± 16	0.396
Laboratory data [median (IQR)]				
Blood glucose level (mg/dL)	129.6 (111.6–158.4)	131.4 (111.6–162.0)	127.8 (109.8–151.2)	0.234
WBC (^*^10^9^/L)	7.76 (6.43–9.78)	7.83 (6.43–9.89)	7.63 (6.36–9.26)	0.411
NLR	3.48 (2.16–5.80)	3.64 (2.19–6.40)	3.20 (2.13–4.93)	0.199
Platelet (^*^10^9^/L)	210 (174–249)	210 (174–254)	210 (174.5–246.5)	0.924
PT (s)	12.9 (12.3–13.5)	12.9 (12.3–13.4)	12.9 (12.3–13.5)	0.984
APTT (s)	34.0 (31.7–36.5)	33.8 (31.6–36.1)	34.5 (31.9–36.9)	0.218
Fibrinogen (g/L)	3.31 (2.81–3.78)	3.34 (2.84–3.79)	3.21 (2.78–3.73)	0.247
Uric acid (μmol/L)	331.0 (275.6–388.6)	328.8 (275.9–383.5)	336.4 (274.2–400.5)	0.317
AGR, median (IQR)	1.50 (1.34–1.65)	1.49 (1.32–1.63)	1.52 (1.36–1.74)	0.181
Triglycerides (mmol/l)	1.51 (1.06–1.92)	1.52 (1.03–1.91)	1.49 (1.06–1.94)	0.750
HDL (mmol/l)	1.14 (0.96–1.32)	1.13 (0.96–1.32)	1.14 (0.96–1.31)	0.814
LDL (mmol/l)	2.85 (2.25–3.33)	2.89 (2.25–3.35)	2.83 (2.25–3.31)	0.463

OTT, onset-to-treatment time; NIHSS, National Institutes of Health Stroke Scale; SBP, systolic blood pressure; DBP, diastolic blood pressure; WBC, white blood cell; NLR, neutrophil-to-lymphocyte ratio; PT, prothrombin time; APTT, activated partial thromboplastin time; AGR, albumin-to-globulin ratio; HDL, high-density lipoprotein; LDL, low-density lipoprotein; IQR, interquartile range; SD, standard deviation.

* Stands for × in the mathematical notation.

As shown in Table 2, 45 (17.5%) were post-thrombolysis HT in the training cohort. The univariate analysis revealed that atrial fibrillation, early infarct signs, current use of antiplatelet agents, NIHSS scores, NLR, PT, fibrinogen, uric acid, and AGR were related to HT (*P* < 0.05). No significant statistical collinearity was observed among the nine variables (Supplementary Table 1). After multivariate logistic regression analysis, the early infarct signs (OR, 7.954; 95% CI, 3.553–17.803; *P* < 0.001), NIHSS scores (OR, 1.110; 95% CI, 1.054–1.168; *P* < 0.001), uric acid (OR, 0.993; 95% CI, 0.989–0.997; *P* = 0.001), and AGR (OR, 0.109; 95% CI, 0.023–0.508; *P* = 0.005) were independent predictors for HT after intravenous thrombolysis in patients with ischemic stroke. In the validation cohort, we found significant differences in the early infarct signs, NIHSS scores, uric acid, and AGR between the HT and non-HT groups (Supplementary Table 2).

**Table 2:** Baseline characteristics and logistic regression analysis between the HT group and non-HT group in the training cohort.

**Variable**	**HT (*n =* 45)**	**Non-HT (*n =* 212)**	**Univariate analysis**		**Multivariate analysis**	
			**OR (95%CI)**	***P*-value**	**OR (95%CI)**	***P*-value**
**Demographic data**						
Age	69 (59–76)	66 (55–73)	1.022 (0.995–1.049)	0.109		
Female	21 (46.7)	72 (34.0)	0.588 (0.307–1.127)	0.110		
**Vascular risk factors**						
Hypertension	32 (71.1)	144 (67.9)	1.162 (0.574–2.355)	0.676		
Atrial fibrillation	22 (48.9)	52 (24.5)	2.943 (1.517–5.711)	**0.001**	NA	0.073
Diabetes mellitus	11 (24.4)	51 (24.1)	1.021 (0.483–2.161)	0.956		
Hyperlipidemia	3 (6.7)	19 (9.0)	0.726 (0.205–2.564)	0.618		
Previous stroke	7 (15.6)	31 (14.6)	1.076 (0.441–2.623)	0.873		
History of smoking	16 (35.6)	86 (40.6)	0.808 (0.414–1.578)	0.533		
History of drinking	7 (15.6)	32 (15.1)	1.036 (0.426–2.522)	0.938		
Antiplatelet agents	7 (15.6)	12 (5.7)	3.070 (1.136–8.301)	**0.027**	NA	0.294
Anticoagulants	9 (20.0)	28 (15.1)	1.406 (0.618–3.198)	0.416		
**Baseline data**						
OTT	182 (130–242)	180 (140–230)	1.002 (0.997–1.006)	0.523		
Early infarct signs	28 (62.2)	39 (18.4)	7.306 (3.644–14.648)	**<0.001**	7.954 (3.553–17.803)	**<0.001**
NIHSS scores	14 (11–18)	6 (3–10)	1.122 (1.070–1.176)	**<0.001**	1.110 (1.054–1.168)	**<0.001**
SBP	159 ± 22	155 ± 26	1.007 (0.994–1.019)	0.309		
DBP	89 ± 14	88 ± 15	1.004 (0.982–1.025)	0.741		
**Laboratory data**						
Blood glucose level	142.2 (120.6–181.8)	129.6 (111.6–159.7)	1.003 (0.999–1.008)	0.162		
WBC	8.47 (6.50–10.50)	7.72 (6.43–9.75)	1.042 (0.957–1.135)	0.342		
NLR	5.44 (2.99–9.46)	3.36 (2.16–5.53)	1.057 (1.000–1.117)	0.048	NA	0.886
Platelet	197 (164–235)	212.5 (177.0–255.7)	0.997 (0.991–1.002)	0.199		
PT	13.5 (12.7–14.5)	12.8 (12.3–13.2)	1.215 (1.002–1.473)	0.048	NA	0.408
APTT	33.4 (30.2–37.9)	33.8 (31.7–36.0)	1.039 (0.991–1.090)	0.116		
Fibrinogen	3.49 (2.90–4.32)	3.32 (2.81–3.75)	1.577 (1.125–2.208)	**0.008**	NA	0.587
Uric acid	379.0 (202.8–371.1)	333.8 (289.2–383.9)	0.994 (0.990–0.998)	**0.002**	0.993 (0.989–0.997)	**0.001**
AGR	1.37 (1.14–1.61)	1.51 (1.36–1.64)	0.088 (0.022–0.344)	**<0.001**	0.109 (0.023–0.508)	**0.005**
Triglycerides	1.21 (0.85–1.62)	1.55 (1.07–1.97)	0.731 (0.498–1.073)	0.109		
HDL	1.09 (0.95–1.31)	1.15 (0.96–1.32)	0.369 (0.108–1.256)	0.111		
LDL	2.67 (1.85–3.21)	2.89 (2.31–3.38)	0.737 (0.510–1.065)	0.104		

HT, hemorrhagic transformation; OTT, onset-to-treatment time; NIHSS, National Institutes of Health Stroke Scale; SBP, systolic blood pressure; DBP, diastolic blood pressure; WBC, white blood cell; NLR, neutrophil-to-lymphocyte ratio; PT, prothrombin time; APTT, activated partial thromboplastin time; AGR, albumin-to-globulin ratio; HDL, high-density lipoprotein; LDL, low-density lipoprotein; IQR, interquartile range; SD, standard deviation; NA, not available. The bold values means *P* < 0.05.

**Figure 2:**
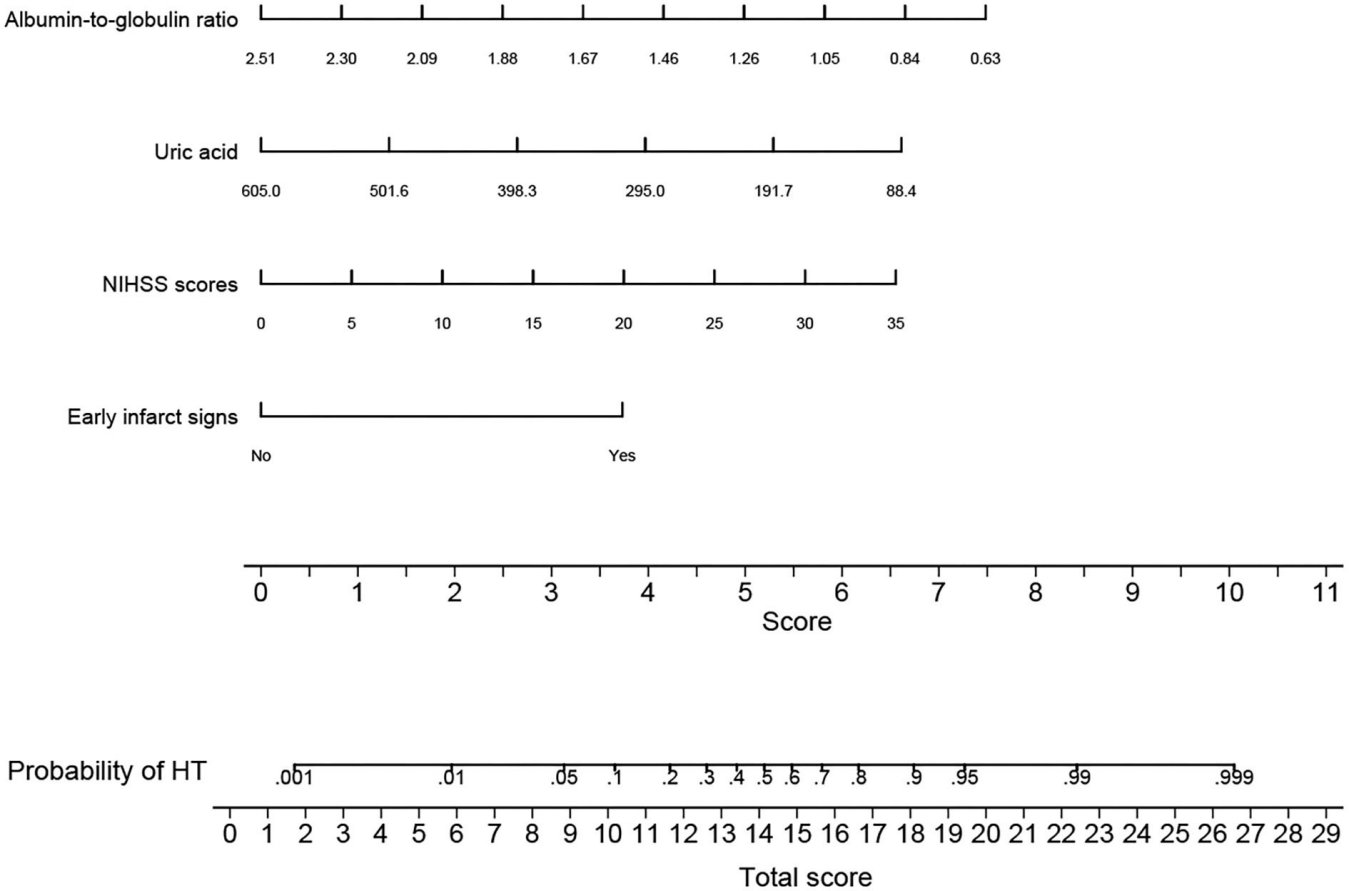
Nomogram for predicting HT after intravenous thrombolysis. The nomogram consists of four predictors, each of which is given a preliminary score (0 – 11). The total scores were obtained by summing all the preliminary scores of each of the four predictors. The estimated probability of hemorrhagic transformation was obtained from the nomogram according to the total score. For example, a patient with an early infarct sign, baseline NIHSS scores of 10, a uric acid level of 295 μmol/L, and an albumin-to-globulin ratio of 1.05 would have a total of 15.7 scores. The probability of HT after intravenous thrombolysis was approximately 70% for the patient. NIHSS, National Institute of Health Stroke Scale; HT, hemorrhagic transformation; CT, computed tomography.

All independent predictors for HT after intravenous thrombolysis were used to construct the novel nomogram (Figure 2). The nomogram consisted of the preliminary value of predictors, preliminary score range (0–11), total score, and probability of HT. Drawing a line downward from the preliminary value to the corresponding preliminary score, and then summed all the preliminary scores to obtain a total score. Finally, the percentage corresponding to the total score was the individual probability of HT after intravenous thrombolysis.

**Figure 3:**
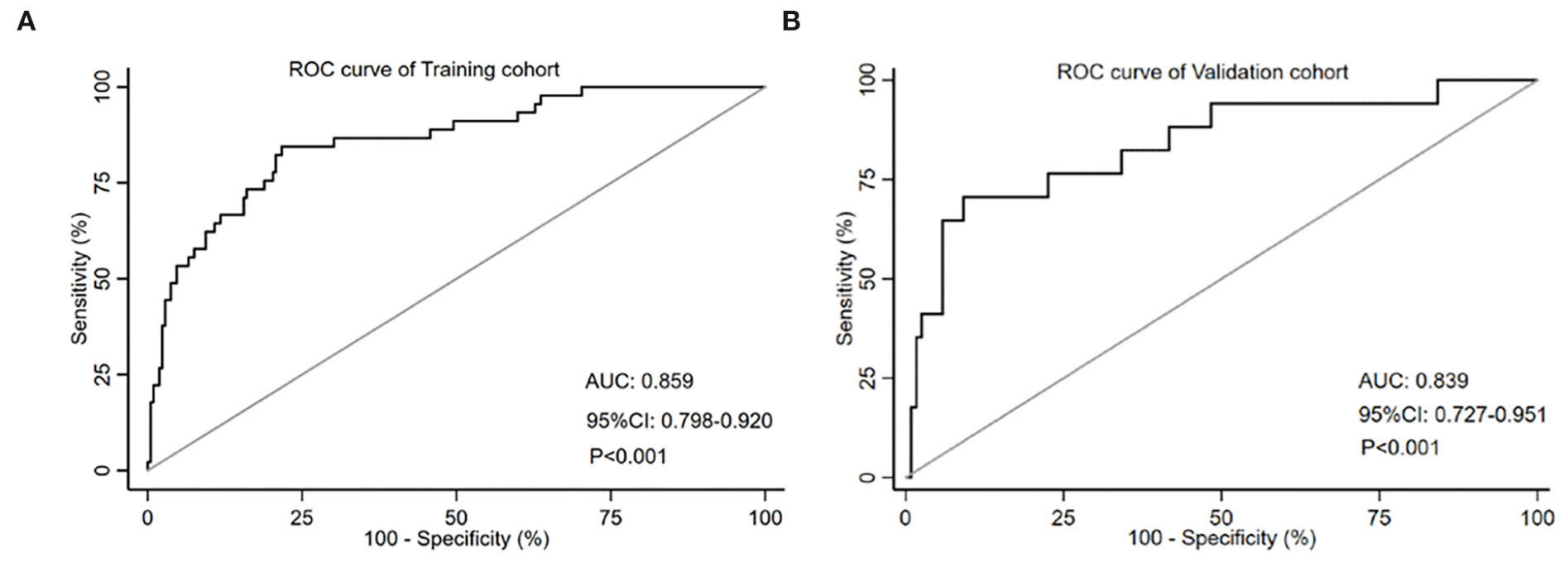
The ROC curve of the nomogram for predicting HT in the training cohort **(A)** and the validation cohort **(B)**. The AUC value is 0.859 in the training cohort and 0.839 in the validation cohort. HT, hemorrhagic transformation; ROC, receiver operating characteristic; AUC, the area under curve.

**Figure 4:**
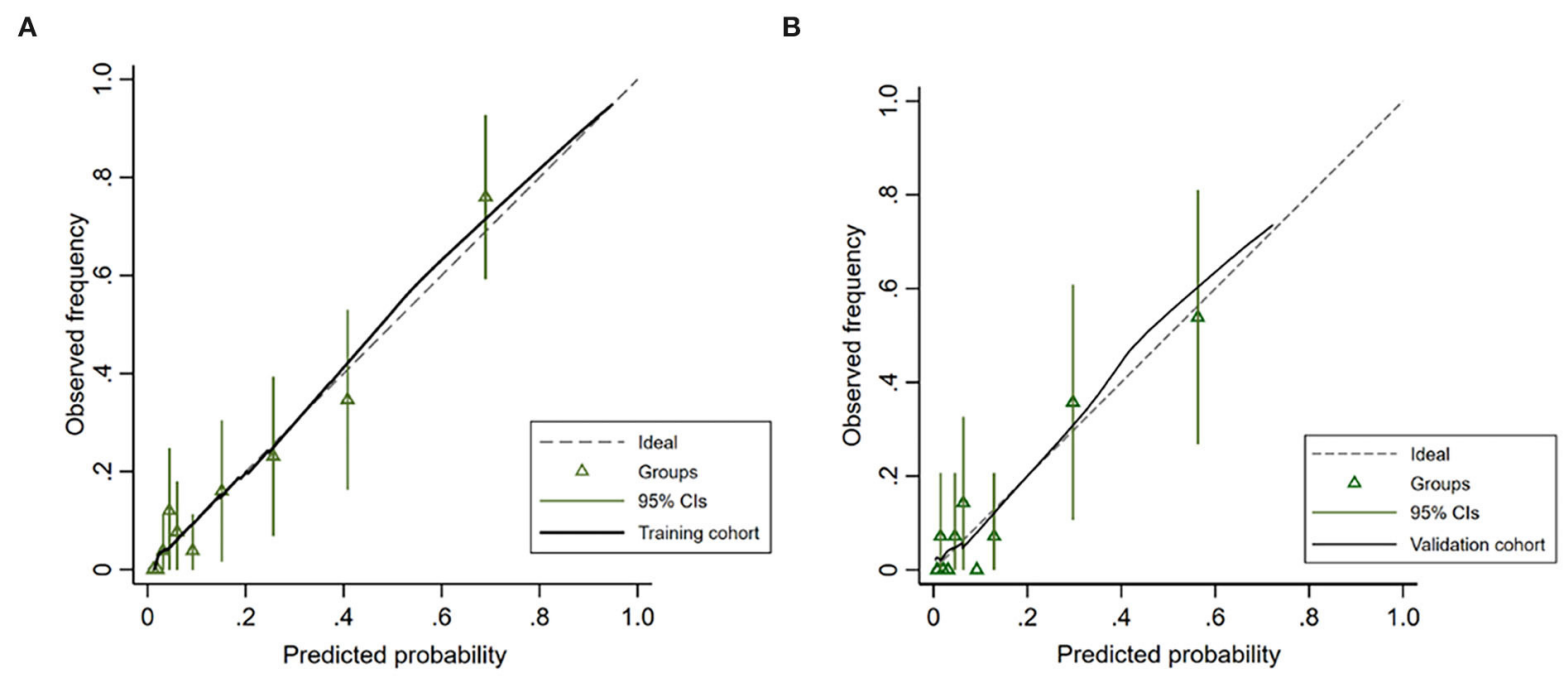
Calibration plot for predicting HT after intravenous thrombolysis in the training cohort **(A)** and the validation cohort **(B)**.

The AUC-ROC was used to evaluate the discriminative ability of the nomogram, which demonstrated a moderate predictive power in the training cohort (AUC, 0.859; 95% CI, 0.798–0.920) (Figure 3A) and validation cohort (AUC, 0.839; 95% CI, 0.727–0.951) (Figure 3B). The Hosmer–Lemeshow goodness-of-fit test showed good concordance between predicted and observed probability for the training cohort (χ^2^ = 6.213, *df* = 8, *P* = 0.623) and the validation cohort (χ^2^ = 9.668, *df* = 8, *P* = 0.289). The calibration plot also revealed significant predictive accuracy of the nomogram to predict HT after intravenous thrombolysis in the training (Figure 4A) and validation cohorts (Figure 4B).

**Figure 5:**
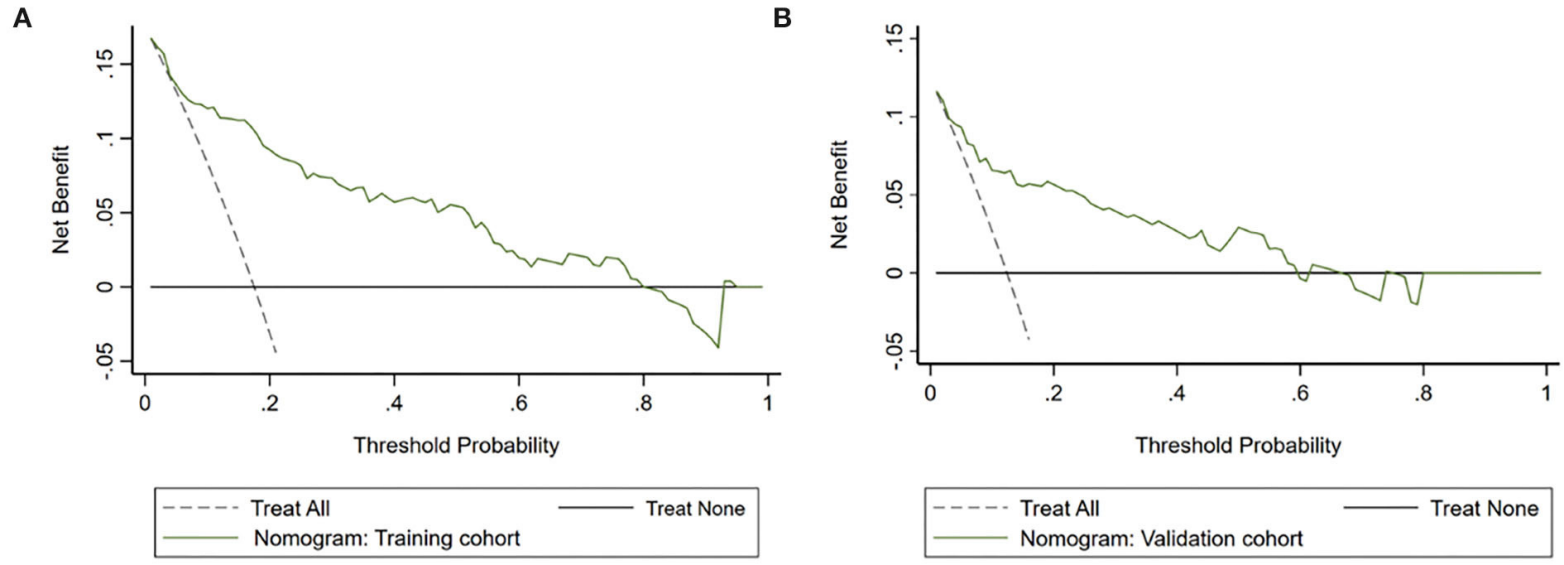
Decision curve analysis (DCA) of the nomogram predicting HT after intravenous thrombolysis in the training cohort **(A)** and the validation cohort **(B)**. The *x*-axis demonstrates the threshold probability. The *y*-axis indicates the net benefit. The black line displays all patients are negative and have no treatment, the net benefit is zero. The dotted line means all patients who accept intravenous thrombolysis will develop HT. The green line indicates the net benefit of the nomogram.

The DCA demonstrated that the novel nomogram had a greater net benefit to predict HT than “treat all” or “treat none” strategies when the threshold probabilities ranged between 5.0% to 80.0% in the training cohort (Figure 5A) and between 3.0% and 60.0% in the validation cohort (Figure 5B), which indicated the good clinical validity of the nomogram in the training and validation cohorts.

## Discussion

In this retrospective single-center study, we presented and validated a practical nomogram based on four predictors including early infarct signs, NIHSS scores, uric acid, and albumin-to-globulin ratio (AGR), which is considered a reliable visual scoring system for predicting HT after intravenous thrombolysis in patients with ischemic stroke. All of these predictors are easily and quickly obtainable before or during treatment. The overall predictive performance of the nomogram was well in the training cohort (AUC-ROC, 0.859) and validation cohort (AUC-ROC, 0.839), which can help neurologists identify ischemic stroke patients who have a higher risk of developing HT after intravenous thrombolysis. Our nomogram has an excellent calibration capability due to the predicted risk for HT being close to the actual risk both in the training cohort, and further confirmed in the validation cohort. Finally, the decision curve analysis (DCA), a special tool to evaluate the clinical application value of a nomogram, suggested that our nomogram was very useful for predicting post-thrombolysis HT in clinical practice.

To identify the probability of post-thrombolysis HT in patients with ischemic stroke, several prediction models have been established in recent years (6–9). Consistent with these previous studies, it was found that NIHSS scores and early infarct signs were conventional predictors for HT in patients who were undergoing rt-PA intravenous thrombolysis. Uric acid, one of the most important endogenous antioxidants, is the final product of purine metabolism that plays a neuroprotective role by scavenging free radicals, inhibiting neuroinflammatory cascades, and reducing the blood-brain barrier permeability (20–22). Previous studies indicated that a lower uric acid level was independently associated with a high risk of HT after intravenous thrombolysis (23, 24). Our study also found that lower uric acid could increase the risk of HT after intravenous thrombolysis.

Notably, our study ascertained that AGR might be a protective factor for post-thrombolysis HT in patients with ischemic stroke. Few previous studies have reported this conclusion. Serum albumin is a multifunctional protein that is synthesized in the liver. Albumin has been shown to have antioxidant, anti-inflammation, and anti-apoptosis in endothelial cells effects (25). Previous studies have shown that the decrease in albumin level or increase in globulin level might be associated with post-thrombolysis HT in ischemic stroke patients (26, 27). Our study also showed that serum AGR (*P* < 0.001) was related to post-thrombolysis HT in univariate analysis. After multivariate analysis, the AGR was an independent predictor for post-thrombolysis HT, which had not been reported in previous studies. We speculated that the possible mechanism of post-thrombolysis HT is the result of the combined influence of albumin and globulin. Lower AGR indicates the decrease in serum albumin or the increase in globulin, which is significantly associated with the increased occurrence of HT after intravenous thrombolysis.

Yet we failed to find the relationship between atrial fibrillation and post-thrombolysis HT in contrast to previous studies (14, 15, 28). In the present study, the difference in atrial fibrillation was statistically significant in univariate analysis. After multivariable adjustment, no significant statistical difference was observed between HT and atrial fibrillation. We also failed to find the relationship between blood glucose and post-thrombolysis HT in contrast to previous studies (6, 8, 14, 15). In addition, previous clinical studies reported that higher fibrinogen level was significantly related to the occurrence of post-thrombolysis HT in acute ischemic stroke (29, 30). In our study, fibrinogen was a risk factor for HT after intravenous thrombolysis. However, the relationship between fibrinogen and HT did not exist after adjusting for confounding factors. Therefore, atrial fibrillation, blood glucose, and fibrinogen were not included in our final nomogram.

There are several limitations to our study that should be considered. First, it was a single-center retrospective cohort study with a small sample size. We only include variables showing a *P*-value <0.05 in the univariate analysis as candidates for the multivariate regression analysis to improve the statistical power of our results. Second, this nomogram was not validated in external cohorts. Therefore, the multicenter prospective study should be established to validate the applicability of our nomogram before applying it in clinical practice. Third, although we controlled for many variables in establishing our prediction model, we cannot rule out some unmeasured baseline variables (including microbleed, glycosylated hemoglobin, homocysteine, and so on) that may influence the development of HT after intravenous thrombolysis. Future prospective studies should further evaluate whether combining with other variables can help to enhance the accuracy of our nomogram prediction. In addition, another limitation is that the uric acid and albumin to globulin ratio could not be obtained before intravenous thrombolysis in some stroke centers, which may limit the application of the nomogram before intravenous thrombolysis in these centers. Due to the narrow time window for the treatment of ischemic stroke, the emergency green channel has been widely opened in many countries to ensure that patients with ischemic stroke can receive intravenous thrombolysis quickly and benefit from it. According to the Chinese guideline, all patients with ischemic stroke should receive NCCT, blood glucose, and laboratory tests including uric acid and albumin to globulin ratio before intravenous thrombolysis. With the establishment of the green channel, uric acid and albumin to globulin ratio can be obtained before intravenous thrombolysis in most stroke centers in our country. Therefore, the nomogram may have good clinical application in our country. However, clinicians in other countries cannot use our nomogram to assess the risk of post-thrombolysis HT in patients with acute ischemic stroke without obtaining the availability of laboratory test results before intravenous thrombolysis. But it could help clinicians assess the probability of HT in patients who are receiving intravenous thrombolysis. For patients with a high risk of post-thrombolysis HT by the evaluation of our nomogram, clinicians may consider using a lower concentration of alteplase or discontinuing intravenous thrombolysis to improve the safety of intravenous thrombolysis.

## Conclusions

Our study proposes a novel and practical nomogram based on early infarct signs, NIHSS scores, uric acid, and albumin-to-globulin ratio that can well predict the probability of HT after intravenous thrombolysis in patients with ischemic stroke. The calibration and discrimination of the nomogram were verified in internal validation. This nomogram can be useful for predicting the probability of HT after intravenous thrombolysis, and help clinicians assess whether to continue intravenous thrombolysis in patients with a high risk of HT. However, further studies are needed to confirm the validity of the nomogram.

## Data availability statement

The raw data supporting the conclusions of this article will be made available by the authors, without undue reservation.

## Ethics statement

The studies involving human participants were reviewed and approved by the Ethics Committee of the Second Xiangya Hospital of Central South University. Written informed consent for participation was not required for this study in accordance with the national legislation and the institutional requirements.

## Author contributions

MY performed the data collection, data analysis, and wrote the manuscript. JP performed the literature search. WZo helped with data analysis. WZh and XT reviewed and edited the manuscript. XT takes responsibility for the integrity of the work from its inception to publishing. All authors read and approved the final manuscript.

## Funding

This study was supported by the Natural Science Foundation of Hunan Province, China [Grant Number 2022JJ30841].

## Conflict of interest

The authors declare that the research was conducted in the absence of any commercial or financial relationships that could be construed as a potential conflict of interest.

## Publisher's note

All claims expressed in this article are solely those of the authors and do not necessarily represent those of their affiliated organizations, or those of the publisher, the editors and the reviewers. Any product that may be evaluated in this article, or claim that may be made by its manufacturer, is not guaranteed or endorsed by the publisher.
