# Supplementary material for: A novel nomogram to predict hemorrhagic transformation in ischemic stroke patients after intravenous thrombolysis
Source: Front Neurol. 2022 Sep 8;13:913442. doi: 10.3389/fneur.2022.913442 (PMC9494598; doi:10.3389/fneur.2022.913442)
Supplement: Supplementary file 1 [file Table_1.docx]

**Supplementary Table 1**. Collinearity of combinations of variables in the training cohort.

| Variable | Tolerance | Variation inflation factor |
| --- | --- | --- |
| Atrial fibrillation | 0.837 | 1.075 |
| Early infarct signs | 0.930 | 1.194 |
| Antiplatelet agents | 0.948 | 1.054 |
| NIHSS scores | 0.825 | 1.213 |
| NLR | 0.825 | 1.211 |
| PT | 0.878 | 1.138 |
| Fibrinogen | 0.817 | 1.224 |
| Uric acid | 0.912 | 1.097 |
| AGR | 0.814 | 1.228 |

All values of tolerance were > 0.2 and variation inflation factor were < 5. Abbreviations: NIHSS, National Institutes of Health Stroke Scale; NLR, neutrophil-to-lymphocyte ratio; PT, prothrombin time; AGR, albumin-to-globulin ratio.
