# Supplementary material for: A novel nomogram to predict hemorrhagic transformation in ischemic stroke patients after intravenous thrombolysis
Source: Front Neurol. 2022 Sep 8;13:913442. doi: 10.3389/fneur.2022.913442 (PMC9494598; doi:10.3389/fneur.2022.913442)
Supplement: Supplementary file 2 [file Table_2.docx]

**Supplementary Table 2**. Comparison of baseline characteristics of ischemic stroke patients between HT group and non-HT group in the validation cohort.

| Variable | HT (n = 17) | Non-HT (n = 120) | *P*-value |
| --- | --- | --- | --- |
| Demographic data |  |  |  |
| Age (years), median (IQR) | 65 (54-73) | 64 (55-73) | 0.761 |
| Female, n (%) | 7 (41.2) | 43 (35.8) | 0.668 |
| Vascular risk factors, n (%) |  |  |  |
| Hypertension | 8 (47.1) | 81 (67.5) | 0.098 |
| Atrial fibrillation | 8 (47.1) | 38 (31.7) | 0.209 |
| Diabetes mellitus | 5 (29.4) | 28 (23.3) | 0.583 |
| Hyperlipidemia | 1 (5.9) | 9 (7.5) | 0.805 |
| Previous stroke | 3 (17.4) | 15 (12.5) | 0.557 |
| History of smoking | 7 (41.2) | 48 (40.0) | 0.926 |
| History of drinking | 3 (17.6) | 27 (22.5) | 0.764 |
| Baseline data |  |  |  |
| OTT (min), median (IQR) | 180 (139-223) | 170 (130-210) | 0.654 |
| Early infarct signs, n (%) | 10 (58.8) | 26 (21.7) | **0.001** |
| NIHSS scores, median (IQR) | 14 (8-19) | 5 (3-10) | **<0.001** |
| SBP (mmHg), mean ± SD | 154 ± 27 | 152 ± 25 | 0.750 |
| DBP (mmHg), mean ± SD | 88 ± 15 | 87 ± 16 | 0.767 |
| Laboratory data (median (IQR)) |  |  |  |
| Blood glucose level (mg/dL) | 147.6 (110.7-183.6) | 126.9 (109.8-147.6) | 0.152 |
| WBC (*10^9/L) | 8.12 (6.32-11.3) | 7.63 (6.33-9.21) | 0.405 |
| NLR | 3.64 (2.38-6.52) | 3.17 (2.12-4.95) | 0.473 |
| Platelet (*10^9/L) | 212 (178-253) | 210 (173-247) | 0.586 |
| PT (s) | 13.1 (11.8-14.5) | 12.8 (12.3-13.4) | 0.489 |
| APTT (s) | 33.2 (28.7-37.4) | 34.5 (32.2-36.9) | 0.312 |
| Fibrinogen (g/L) | 3.36 (3.09-4.18) | 3.19 (2.75-3.68) | 0.057 |
| Uric acid (μmol/L) | 255 (195.6-376.1) | 341.5 (289.2-403.1) | **0.020** |
| AGR, median (IQR) | 1.36 (1.11-1.60) | 1.54 (1.40-1.75) | **0.017** |
| Triglycerides (mmol/l) | 1.52 (1.22-1.80) | 1.45 (1.04-1.97) | 0.804 |
| HDL (mmol/l) | 1.04 (0.95-1.26) | 1.14 (0.96-1.32) | 0.315 |
| LDL (mmol/l) | 2.67 (1.95-3.17) | 2.84 (2.27-3.35) | 0.304 |

OTT, onset-to-treatment time; NIHSS, National Institutes of Health Stroke Scale; SBP, systolic blood pressure; DBP, diastolic blood pressure; WBC, white blood cell; NLR, neutrophil-to-lymphocyte ratio; PT, prothrombin time; APTT, activated partial thromboplastin time; AGR, albumin-to-globulin ratio; HDL, high-density lipoprotein; LDL, low-density lipoprotein; IQR, interquartile range; SD, standard deviation. The bold means P < 0.05. * stands for × in mathematical notation.
